# The RNA-binding protein LARP1 is dispensable for pancreatic β-cell function and mass

**DOI:** 10.1038/s41598-021-81457-4

**Published:** 2021-01-22

**Authors:** Joao Pedro Werneck-de-Castro, Flavia Leticia Martins Peçanha, Diego Henrique Silvestre, Ernesto Bernal-Mizrachi

**Affiliations:** 1 Division of Endocrinology, Diabetes and Metabolism, Department of Internal Medicine, University of Miami Miller School of Medicine, Miami, FL 33136 USA; 2 Institute of Nutrition Josué de Castro, Federal University of Rio de Janeiro, Rio de Janeiro, 21941090 Brazil; 3 Miami VA Health Care System, Miami, FL 33136 USA

**Keywords:** Endocrine system and metabolic diseases, Diabetes

## Abstract

Mechanistic target of rapamycin complex 1 (mTORC1) deficiency or chronic hyperactivation in pancreatic β-cells leads to diabetes. mTORC1 complexes with La-related protein 1 (LARP1) to specifically regulate the expression of 5′ terminal oligopyrimidine tract (5′TOP) mRNAs which encode proteins of the translation machinery and ribosome biogenesis. Here we show that LARP1 is the most expressed LARP in mouse islets and human β-cells, being 2–4-fold more abundant than LARP1B, a member of the family that also interacts with mTORC1. Interestingly, β-cells from diabetic patients have higher LARP1 and LARP1B expression. However, specific deletion of *Larp1* gene in β-cells (*β-Larp1KO* mice) did not impair insulin secretion and glucose metabolism in male and female mice. High fat or high branched-chain amino acid (BCAA) diets did not disturb glucose homeostasis compared to control littermates up to 8 weeks; BCAA diet slightly impaired glucose tolerance in the *β-Larp1KO* mice at 16 weeks. However, no differences in plasma insulin levels, non-fasting glycemia and β-cell mass were observed in the *β-Larp1KO* mice. In conclusion, LARP1 is the most abundant LARP in mouse islets and human β-cells, and it is upregulated in diabetic subjects. However, genetically disruption of *Larp1* gene did not impact glucose homeostasis in basal and diabetogenic conditions, suggesting no major role for LARP1 in β-cells.

## Introduction

Insulin secreting β-cell failure is a hallmark of diabetes^1^. Although β-cells are competent in adapting to insulin resistance by secreting more insulin and increasing in mass to maintain glucose homeostasis, the high metabolic demand will eventually progress to β-cell exhaustion and a fraction of obese patients will develop diabetes^1,2^. Mechanistic target of rapamycin complex 1 (mTORC1) is a cellular rheostat linking nutrient availability and growth factor signaling to cell metabolism. We have shown previously that mTORC1 is essential to β-cell function and mass^3,4^. Lack of mTORC1 activity specifically in β-cells impairs proliferation and survival^3^. mTORC1 also regulates insulin processing^3–5^ as well as β-cell maturation^6–8^. On the other hand, chronic hyperactivation of mTORC1 in β-cells culminates into β-cell dysfunction and diabetes^9,10^. These findings underscore the importance of mTORC1 signaling in β-cells.

Protein translation depends on an orchestrated assembling of proteins participating in translation initiation, ribosomal recruitment and protein elongation. mTORC1 controls cell size, proliferation, ribosomal biogenesis, protein translation and autophagy by modulating eIF4E-binding proteins (4E-BP1, 2 and 3) and ribosomal protein S6 kinases (S6K1 and 2) and ULK among others^11,12^. It also enhances cellular protein synthesis capacity by inducing translation of certain 5′ terminal oligopyrimidine tract (5′TOP) mRNAs which encode proteins of the translation machinery and ribosome biogenesis^13–16^. mTORC1 inhibition by rapamycin represses TOP mRNA translation^15,17,18^ and the 4EBP proteins have been proposed as suppressors of TOP mRNAs translation^14^. Recently, a downstream target of mTORC1, the La-related protein 1 (LARP1), also known as RNA-binding protein LARP1, has been described as part of the protein complex regulating the 5′-TOP mRNA translation^19–24^. The LARP family consists of six members: LARP1, 2 (1B), 4, 5 (4B), 6, and 7^23,25^. They all contain the RNA-binding domain but only LARP1 and LARP1B present the DM15 motif and interact with mTORC1^23,25^.

The role of LARP1 in protein synthesis and mTORC1-mediated TOP mRNA translation is controversial^26^. Studies in HeLa and HEK293 cells have demonstrated that LARP1 positively regulates protein synthesis^22,27,28^. Knockdown of LARP1 impaired cell division and migration, and induces cell apoptosis as well as a 15% drop in overall protein synthesis accompanied by hypophosphorylated 4E-BP1 levels^28^, indicating participation in cap-dependent translation. LARP1 depletion was associated with a decreased TOP mRNAs translation^22^. In contrast, also using HEK293T and HeLa cells, Fonseca et al. concluded that LARP1 functions as an important repressor of TOP mRNA translation downstream of mTORC1^19^. They showed that LARP1 interacts with TOP mRNAs in an mTORC1-dependent manner and competes with the eIF4G for TOP mRNA binding. Reducing LARP1 protein levels by siRNA attenuates the inhibitory effect of rapamycin and Torin1 on TOP mRNA translation^19^. That LARP1 directly binds the cap and adjacent 5′TOP motif of TOP mRNAs impeding access of eIF4E and eIF4F assembly was confirmed later^24^. Phosphorylation of LARP1 by mTORC1 and Akt/S6K1 dissociates it from 5′UTRs and relieves its inhibitory activity^23^. Concomitantly, phosphorylated LARP1 scaffolds mTORC1 on the 3′UTRs to facilitate mTORC1-dependent induction of translation initiation. Although LARP1 has inhibitory effects on TOP mRNA translation, LARP1 loss of function causes inefficient translation elongation^23^. The in vivo role of LARP proteins has been limited by the lack of animal models to study the tissue specific importance of this molecule.

We document herein that LARP1 is the most abundant of the family in human β-cells and mouse islets by our own experiments and by assessing public RNA expression databases. Interestingly, diabetes increases LARP1 and LARP1B in human β-cells. To study the role of LARP1 in β-cells, we developed mice with conditional inactivation in pancreatic β-cells. We found that *Larp1* gene disruption in mouse β-cells is dispensable for β-cell function and glucose homeostasis during normal conditions and after administration of high fat or high branched-chain amino acid diets. We conclude that although LARP1 is highly expressed in β-cells and is up-regulated in diabetogenic conditions, yet it is not essential for β-cell function and mass, and glucose homeostasis.

## Results

### LARP1 is the most expressed LARP in human and mouse pancreatic islets and β-cells

**Figure 1:**
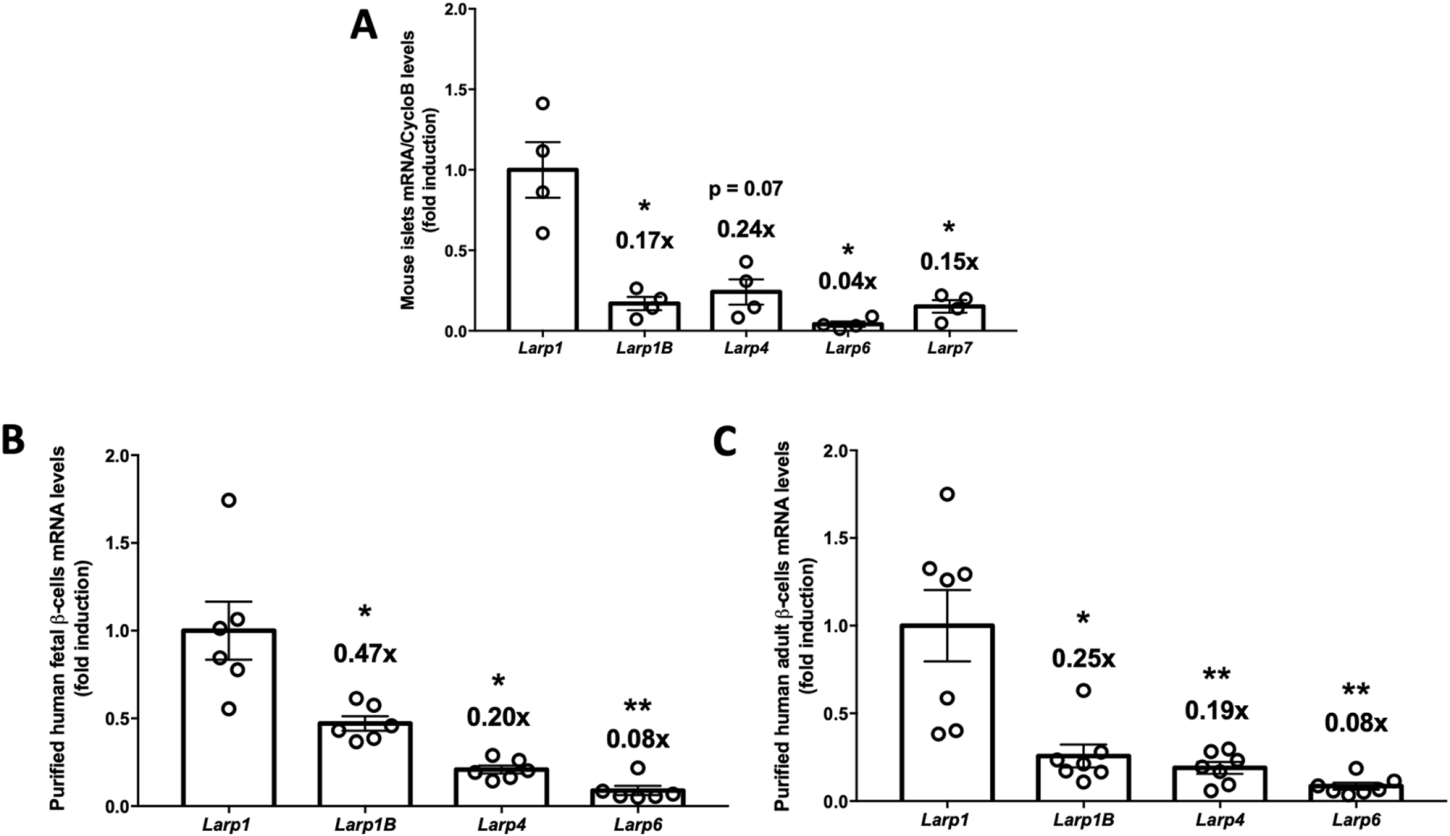
LARP1 is the most expressed LARP in mouse islets and human β-cells. (**A**) LARP family mRNA levels in isolated mouse islets, (**B**) LARP family gene expression in human fetal β-cells and (**C**) Same as in B except that adult human β-cells were used *p < 0.05, **p < 0.01 and ***p < 0.001 compared to LARP1 mRNA levels assessed by one-way analysis of variance (*ANOVA*) followed by Dunnet’s posthoc test. Numbers on top of the bars denote fold reduction. Figures B and C are analysis of RNA sequencing publicly available database by Blodgett et al.^29^.

**Figure 2:**
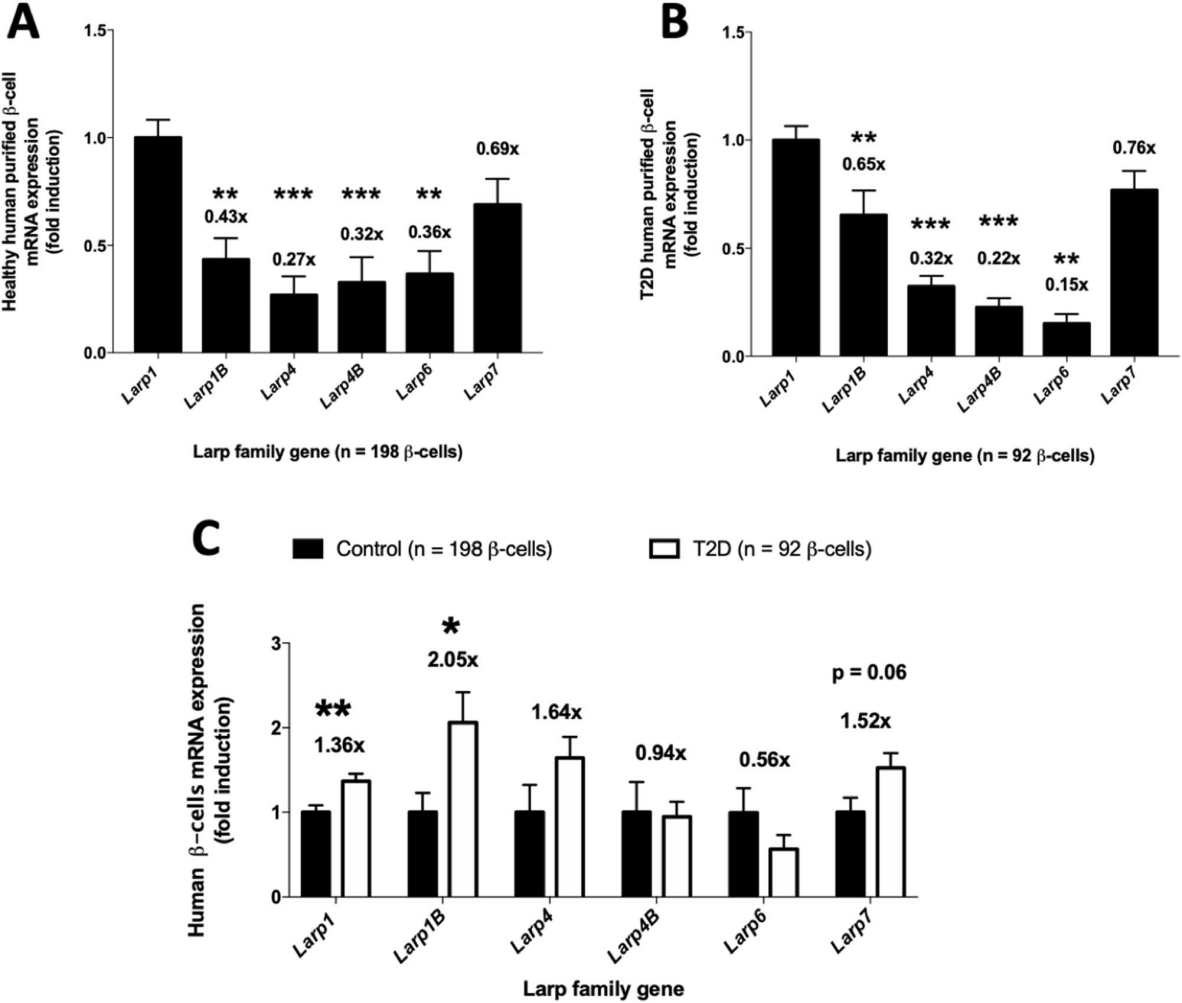
Diabetes increases Larp1 and Larp1B expression in human β-cells. (**A**) LARP family mRNA levels in human β-cells of healthy subjects. (**B**) Same as in A except that human β-cells from type 2 diabetic patients were used. **p < 0.01 and ***p < 0.001 compared to LARP1 mRNA levels assessed by one-way analysis of variance (*ANOVA*) followed by Dunnet’s posthoc test. (**C**) Fold induction of LARP family mRNA levels in β-cells from type 2 diabetes compared to healthy control subjects shown in (**A**,**B**). *p < 0.05 and **p < 0.01 compared to healthy subjects assessed by Student’s T test. Numbers on top of the bars denote fold induction. All data were obtained by analysis of single-cell transcriptome available by Segerstolpe et al*.*^30^.

The RNA-binding La-related protein (LARP) family consists of six members namely LARP1, 2 (1B), 4, 5 (4B), 6, and 7^23,25^. LARP1 and LARP1B share a common DM15 motif and are mTORC1 targets. We first measured the mRNA levels of the LARP family in isolated mouse islets (Fig. 1A). Remarkably, LARP1 is the most expressed LARP in mouse islets, being fourfold higher than LARP1B, LARP4 and LARP7 (Fig. 1A). LARP6 is barely detectable, being only 5% of LARP1 expression (Fig. 1A). Assessment of publicly available mRNA transcription databases for human β-cells^29,30^ shows that fetal and adult purified β-cells present higher levels of LARP1 mRNA levels (~ 2-fold) compared to its paralog gene LARP1B (Fig. 1B,C^29^). Single-cell transcriptome profiling of human β-cells in healthy subjects and type 2 diabetes (T2D) further corroborates that LARP1 is the most abundant LARP in β-cells (Fig. 2A) with similar pattern seen in T2D (Fig. 2B). Interestingly, diabetes increases LARP1 (~ 30%) and LARP1B (100%) (Fig. 2C), suggesting higher protein translation capacity, consistent with greater mTORC1 activity in diabetes^9^.

### *β-Larp1KO* mice: in vivo model to assess LARP1 function

**Figure 3:**
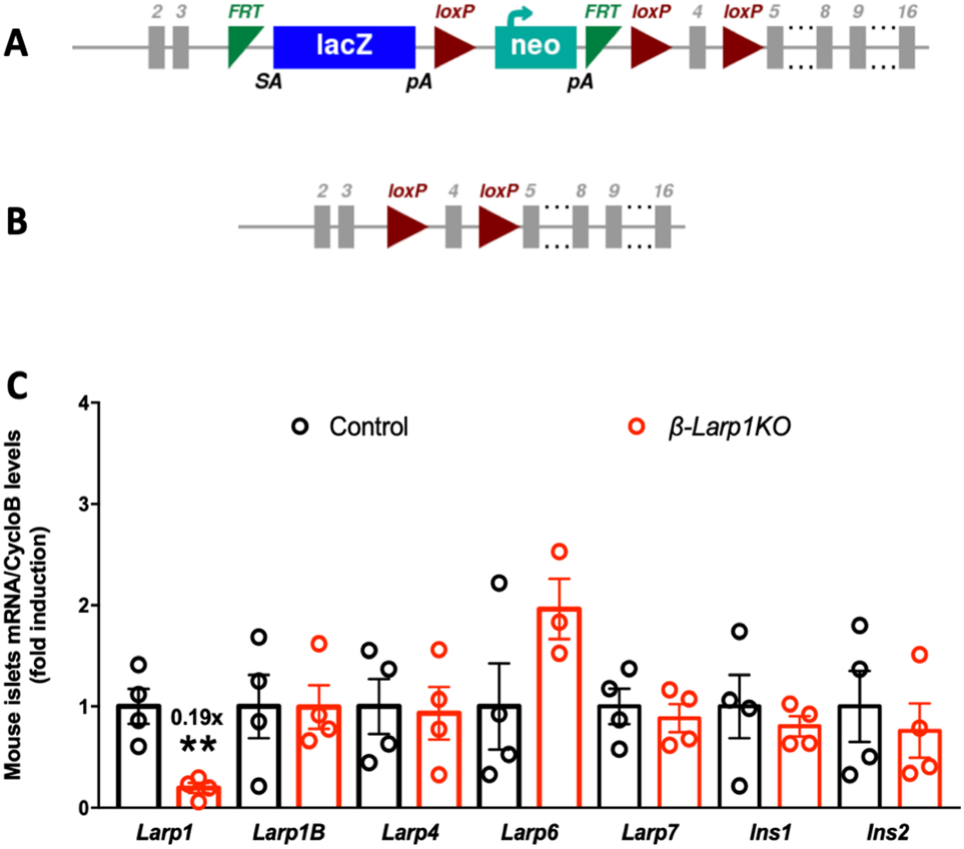
Generation of conditional knockout mice in β-cells (β-Larp1KO). (**A**) *Larp1* gene construct used to generate the *floxed-Larp1* mouse. Mice containing the targeted *Larp1* gene were crossed with a mouse expressing an enhanced variant of Saccharomyces Cerevisiae FLP1 recombinase (FlpE) to remove the lacZ and neomycin-resistance cassettes flanked by the FRT-cutting sites (green), (**B**) *Floxed-Larp1* gene after FLP1 recombination and before Cre-recombinase. After crossing with the Rat insulin promoter (RIP)-Cre mouse, exon 4 is specifically disrupted in β-cells. Grey boxes denote *Larp1* gene exons. (**A**,**B**) were modified from the International Mouse Phenotype Consortium (see link in methods) and (**C**) LARP family and insulin mRNA levels in isolated islets from control and β-Larp1KO mice. ** p < 0.01 compared to control mice assessed by Student’s T test. Number on top of *Larp1* bar denote fold induction.

Whereas the role played by mTORC1 in β- and α-cells was recently revealed^3,4,6,31,32^, the role of the mTORC1 target LARP1 on pancreatic β-cells has never been studied. mTORC1 complexes and phosphorylates LARP1, but whether LARP1 inhibits or stimulates mTORC1-mediated protein translation of TOP mRNAs is still under debate^19,20,22–24,27,28^. Published work has characterized the role of LARP1 using in vitro models. Therefore, we decided to generate a LARP1 deficient mouse specifically in β-cells to evaluate LARP1 function in the context of β-cell biology. We generated a *floxed-LARP1* mouse with the lox p sequence flanking exon 4 (Fig. 3A). After removing the *lacZ* and neomycin-resistance cassettes by crossing with mice expressing FLP1 recombinase (FlpE) under the control of β-actin promoter, *floxed-LARP1* mice were crossed with mice expressing Cre recombinase under the control of the rat insulin promoter to generate the *β-Larp1KO* mouse (Fig. 3B). LARP1 mRNA levels decreased 80% in isolated islets of the *β-Larp1KO* compared to littermate control mice (Fig. 3C). The remaining expression is probably from non-*β*-cells (e.g. α and δ cells) and acinar contaminants. The expected recombination in *β*-cells with the RIP-cre mouse is about 90–95%^3^, therefore, the residual expression can also be explained by the non-recombined *β-*cells. LARP1 and LARP1B are very similar in structure sharing the DM15 region, known as LARP1 motif^23,33^. Therefore, we measured LARP1B and other LARP family members mRNA expression. The deficiency is specific to LARP1, since the *β-Larp1KO* mice have normal expression of LARP1B, LARP4 and LARP7. LARP6 mRNA levels tended to be increased in the *β-Larp1KO* mice but did not reach statistical significance (Fig. 3C).

### β-Larp1KO mice grow and age normally and exhibit normal glucose homeostasis

**Figure 4:**
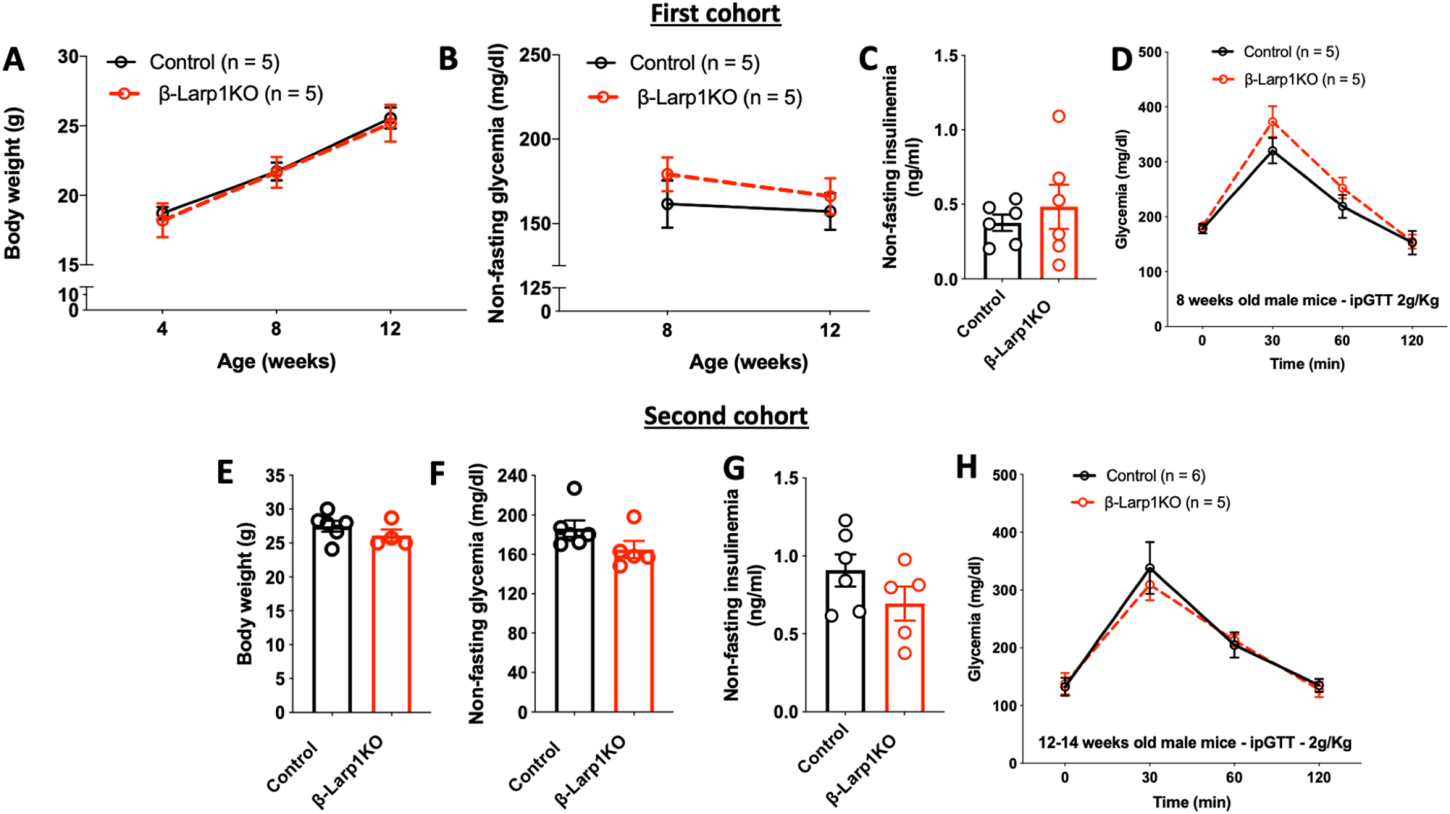
β-Larp1KO mice have normal glucose homeostasis. (**A**) Body weight, (**B**) non-fasting glycemia (**C**) non-fasting insulinemia and (**D**) intraperitoneal glucose tolerance test (ipGTT) in the first cohort of male *β-Larp1KO* mice; (**E**) body weight, (**F**) non-fasting glycemia (**G**) non-fasting insulinemia and (**H**) ipGTT in the second cohort of male *β-Larp1KO* mice.

**Figure 5:**
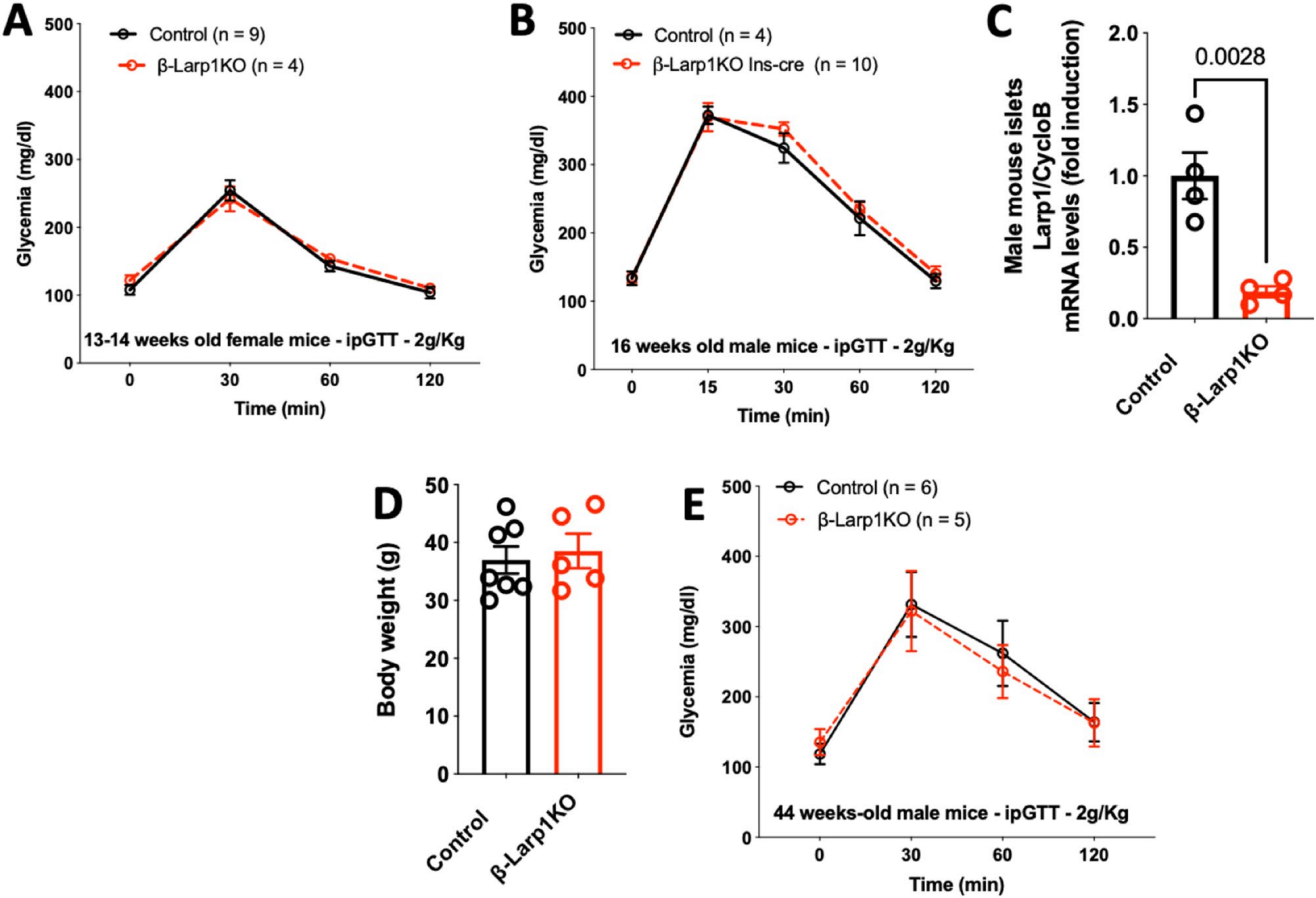
Female β-Larp1KO and old β-Larp1KO male mice are tolerant to glucose as control mice. (**A**) ipGTT in female *β-Larp1KO* mice and (**B**) ipGTT in male *β-Larp1KO* mice generated by crossing the *floxed-Larp1* mice with the *Ins1-cre* mice instead of *Rip-Cre* used in Fig. 4. (**D**) Body weight and (**E**) ipGTT in male *β-Larp1KO* mice at 44 weeks of age. P value shown in (**C**) is compared to control mice assessed by Student’s T test.

We followed up glucose metabolism in *β-Larp1KO* male mice at different ages and gender (Figs. 4, 5). Body weight, non-fasting and fasting glycemia, non-fasting plasma insulin, and tolerance to intraperitoneal glucose load were similar in the first cohort of animals at 4 and 8 weeks of age (Fig. 4A–D). Using a second cohort of *β-Larp1KO* male mice, no difference in body weight, non-fasting and fasting glycemia, non-fasting plasma insulin, and glucose tolerance test was observed at 14 weeks of age (Fig. 4E–H). In addition, there was no difference in glucose tolerance in *β-Larp1KO* female mice (Fig. 5A). We also crossed the *floxed-LARP1* mouse with *Ins1-Cre* knock-in mice (Ins1-cre) and found no difference in glucose tolerance (Fig. 5B) despite the 80% reduction in Larp1 expression by islets (Fig. 5C). To investigate the effects of aging and the lack of LARP1 in *β*-cells, glucose tolerance test was performed in a third cohort of mice aged until almost a year (Fig. 5). Aged *β-Larp1KO* male mice performed similarly to control mice in the glucose tolerance test and weighted equally at 44 weeks of age (Fig. 5D,E).

**Figure 6:**
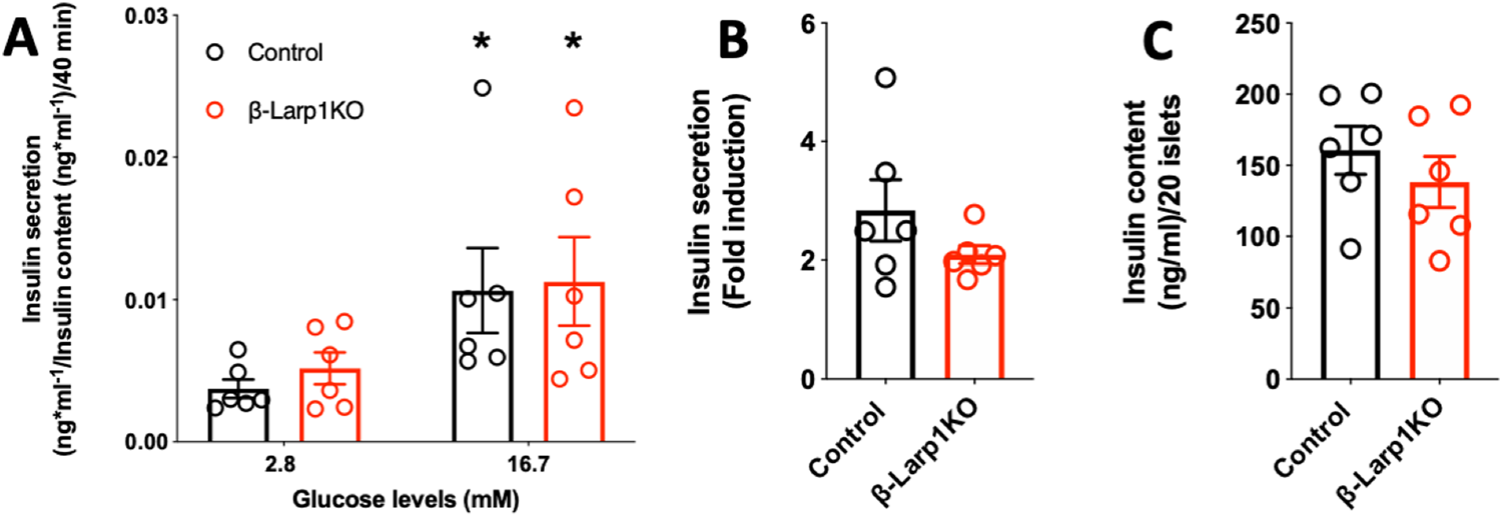
Glucose-stimulated insulin secretion is normal in islets isolated from *β-Larp1KO* *mice.* (**A**) Insulin secretion at low and high glucose levels normalized by insulin content; (**B**) fold induction of insulin secretion by high glucose (16.7 mM) normalized to low glucose (2.8 mM) shown in **A**. (**C**) Insulin content determined in 20 islets. *p < 0.01 compared to 2.8 mM glucose within the group assessed by two-way ANOVA.

To further characterize insulin secretion in the β-Larp1KO mice, we isolated islet from 7-month old male mice to assess GSIS in vitro. Transition from low to high glucose induced insulin secretion equally in both groups (Fig. 6A,B). Importantly, insulin content did not differ from control values (Fig. 6C).

### Exposure of β-Larp1KO mice to high fat diet did not alter glucose homeostasis

**Figure 7:**
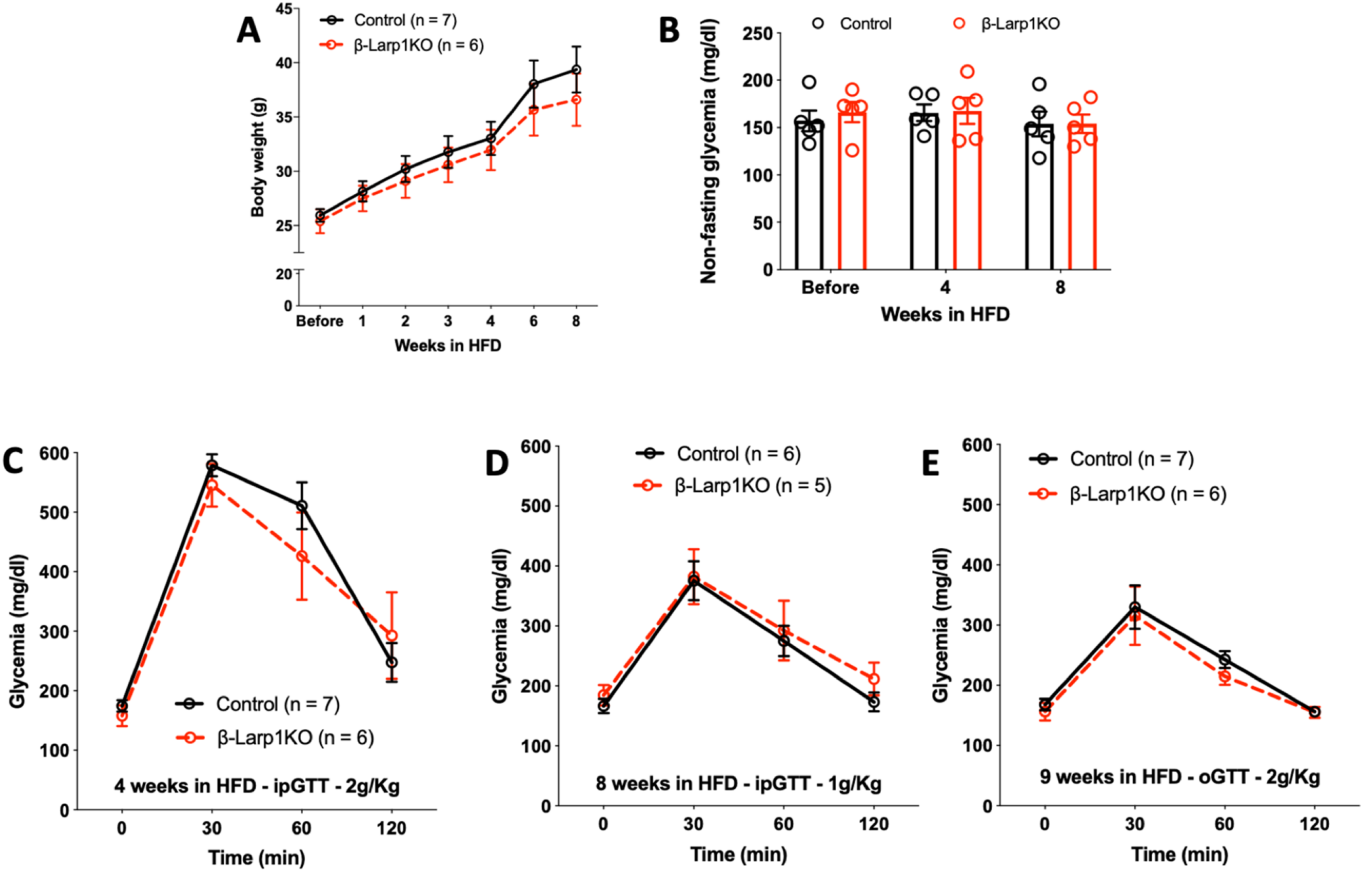
β-Larp1KO mice under high fat diet. (**A**) Body weight, (**B**) non-fasting glycemia of mice fed with high fat diet (60% fat) for 8 weeks. Intraperitoneal glucose tolerance test (ipGTT) after (**C**) 4 and (**D**) 8 weeks in HFD. (**E**) Oral glucose tolerance test (oGTT) after 9 weeks in HF.

It is well-characterized that high fat diet (HFD) provokes insulin resistance, increasing the demand for insulin production and secretion by β-cells, resulting in higher insulinemia. Insulin in turn stimulates β-cell expansion through the activation of Akt/mTORC1 pathway. Therefore, we decided to challenge the *β-Larp1KO* mice under HFD. We placed the first cohort used in Fig. 4A–D under HFD. The *β-Larp1KO* mice gained weigh as much as control mice (Fig. 7A). There was no difference in non-fasting glycemia before after 4 or 8 weeks under HFD (Fig. 7B). Intraperitoneal glucose tolerance test at 4 and 8 weeks after HFD was comparable between *β-Larp1KO* and control mice (Fig. 7C,D). We also tested whether incretins could play a role in the *β-Larp1KO* mice by performing an oral glucose tolerance test and found no difference between the groups (Fig. 7E).

### Long-term exposure to high branched-chain amino acid diet did not impair β-cell function and mass in the β-Larp1KO

**Figure 8:**
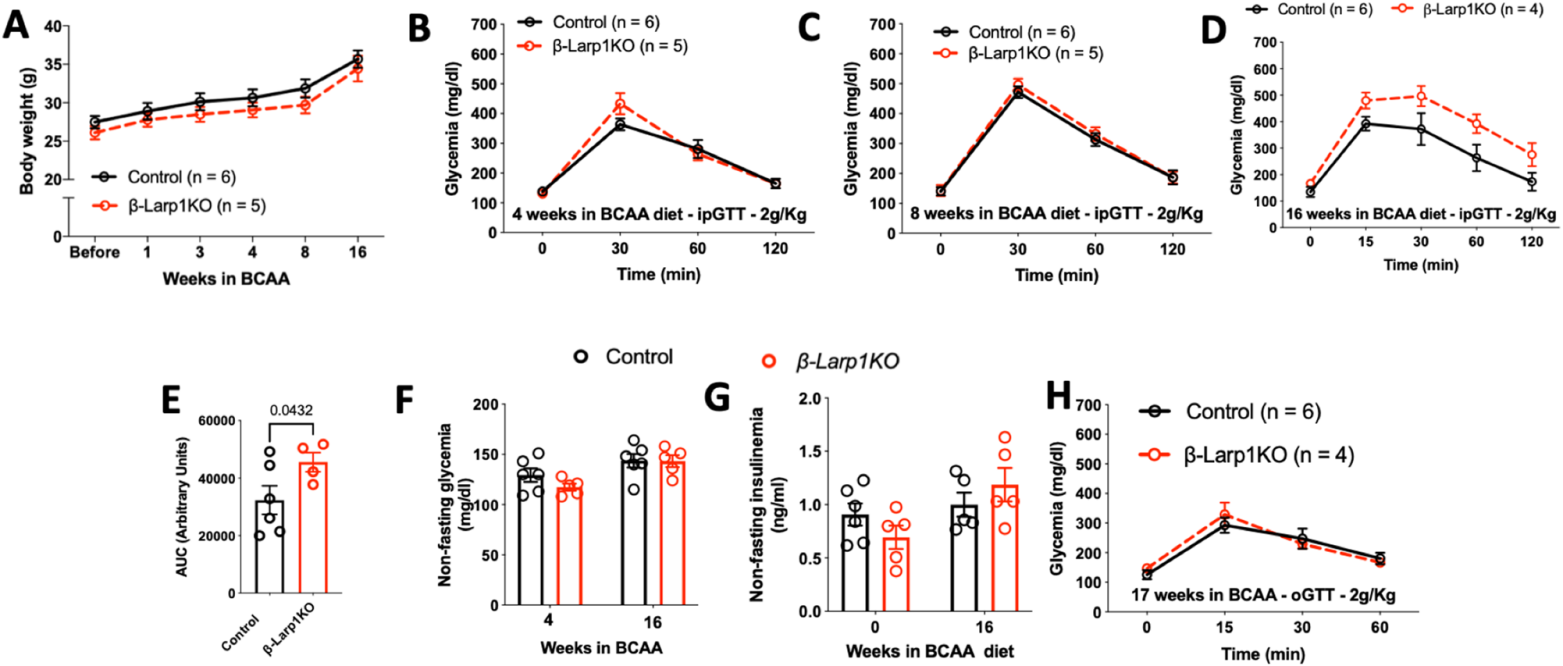
β-Larp1KO mice under high branched-chain amino acid diet (BCAA). (**A**) Body weight of mice fed with high branched-chain amino acid diet (150% BCAA) for 16 weeks. Intraperitoneal glucose tolerance test (ipGTT) after 4 (**B**), 8 (**C**) and 16 (**D**) weeks in BCAA diet. (**E**) Area under the curve in the ipGTT performed in **D**. P value compared to control mice assessed by Student’s T test. Non-fasting glycemia (**F**) and insulinemia (**G**). (**H**) Oral glucose tolerance test (oGTT) after 17 weeks in BCAA diet.

**Figure 9:**
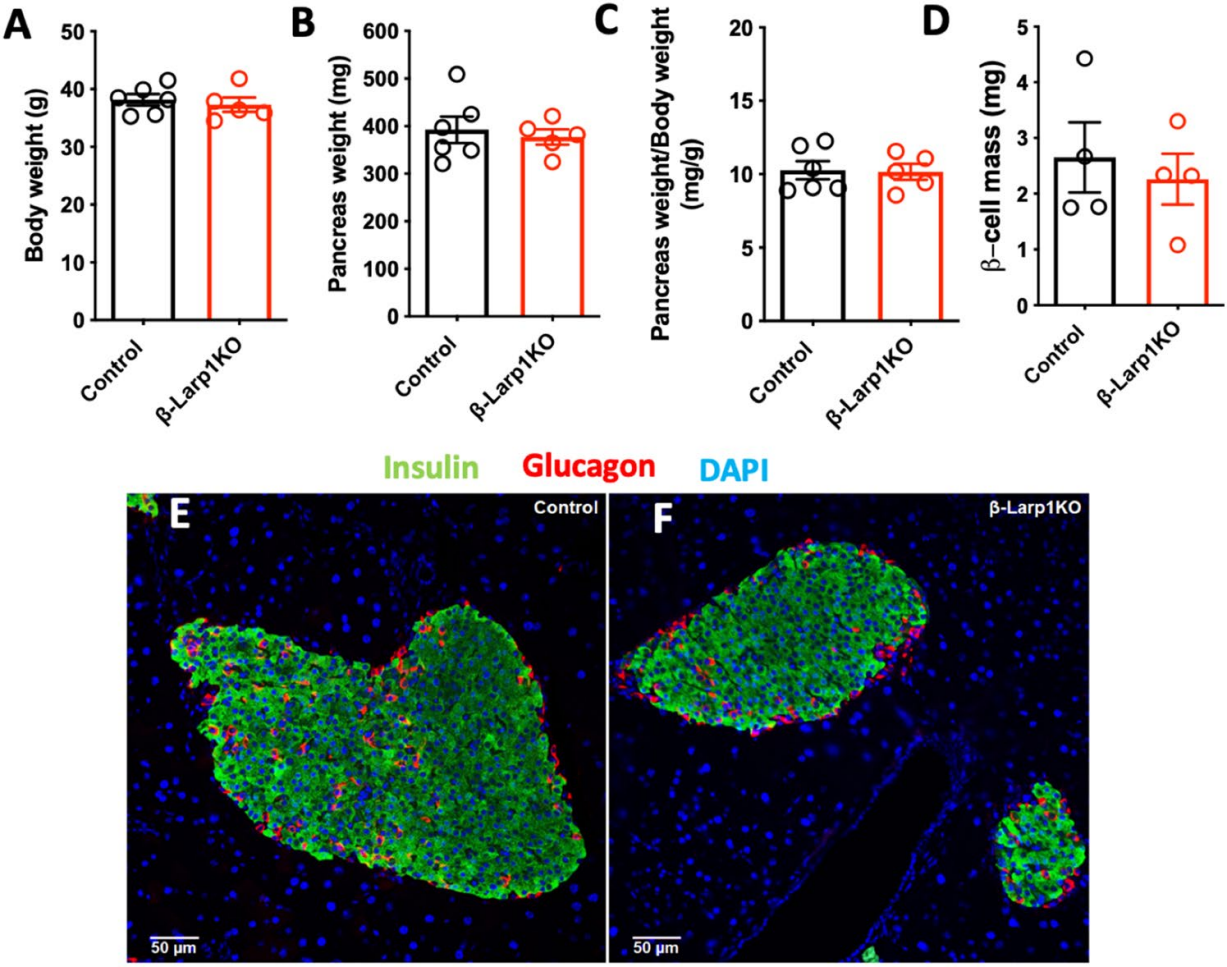
Morphology assessment of β-Larp1KO mice under high branched-chain amino acid diet (BCAA). (**A**) Body weight, (**B**) pancreas absolute weight, (**C**) pancreas weight relative to body weight and (**D**) β-cell mass of mice fed with high branched-chain amino acid diet (150% BCAA) for 17 weeks. (**E**,**F**) Representative images of islets in the control and β-Larp1KO mice, respectively.

As an alternative method, we placed the second-cohort used in Fig. 4E–H under branched-chain amino acids diet (BCAA). BCAA diet directly stimulate mTORC1 activity, especially the enriched l-leucine amino acid^34^. There was no difference in body weight gain in the *β-Larp1KO* and control groups (Fig. 8A). Glucose tolerance was the same between the groups at 4 and 8 weeks after high BCAA diet (Fig. 8B,C). After 16 weeks, *β-Larp1KO* were slightly intolerant (p = 0.04) to glucose compared to littermate control mice when area under the curve was analyzed (Fig. 8D,E). However, non-fasting glycemia and plasma insulin levels were not different between the groups at any time point (Fig. 8F,G). Oral tolerance to glucose was the same after 17 weeks in BCAA diet (Fig. 8H). Histological assessment demonstrated that LARP1 deficiency did not alter pancreas weight and β-cell mass (Fig. 9A–D). In addition, no difference in islet structure between the groups was observed (Fig. 9E,F). Overall, these data suggest that even long-term (> 8 weeks) BCAA diet does not impact beta-cell function, mass and islet morphology.

## Discussion

We report herein that La related protein 1 (LARP1) is highly expressed in human β-cells and mouse islets compared to the other members of the family. Furthermore, type 2 diabetes up-regulates LARP1 and LARP1B. However, mice lacking LARP1 specifically in β-cells (*β*-*Larp1KO* mice) developed and aged normally, and glucose metabolism was similar to control mice. Even under diet-induced stress (high fat or high branched-chain amino acid), glucose metabolism in *β*-*Larp1KO* mice did not deviate from control mice. There was only a minor impaired glucose tolerance after long-term (16 weeks) in BCAA diet but insulin secretion and β-cell mass were normal. Therefore, we conclude that LARP1 is dispensable for pancreatic β-cell function and glucose homeostasis.

A constant cellular regulation of protein synthesis and breakdown determine cellular function and growth. During the progression of diabetes, β-cells expand in size and number to meet the high metabolic demand imposed by insulin resistance^1^. mTORC1 signaling pathway is highly activated in β-cells from diabetic patients and rodents, indicating enhanced protein synthesis and increased proliferation^35^. The current studies were designed to interrogate the in vivo role of LARP1 in β-cell function based on previous finding that LARP1 complexes with mTORC1 and regulates protein translation^23,24,36^. We found that LARP1 is the most expressed LARP in mouse islets and human β-cells. Moreover, LARP1 is up-regulated in β-cells in patients diagnosed with type 2 diabetes. This is suggestive that LARP1 could be required for the high metabolic demand during the development of diabetes because LARP1 regulates proliferation and protein translation^22,23,26^. Moreover, in several different types of cancer, conditions where cell survival and proliferation are enhanced, highly phosphorylated LARP1 has been reported. However, other kinases other than mTORC1 could also induce LARP1 phosphorylation in cancer^26,37–39^.

Surprisingly, we found that *β*-*Larp1KO* mice exhibit a normal phenotype. mTORC1 plays a fundamental role in β-cell physiology by controlling 5′ cap-dependent translation of critical proteins in β-cell^3,6^. mTORC1 activity in β-cells is higher during embryological development and in the first week of post-natal maturation, followed by lower activity levels in mature β-cells^6–8,32,40^. mTORC1 is reactivated in diabetic states and the chronic hyperactivation could play a role in β-cells dysfunction or failure^9,35^. The fact that mTORC1 and LARP1 interact to each other to control cellular protein translation capacity, and, more importantly, that mTORC1 activity and LARP1 expression are both increased in diabetes prompted us to generate a conditional mouse strain to disrupt LARP1 specifically in β-cells. The reasons for the normal glucose metabolism in the *β*-*Larp1KO* mice are not clear. We observed an 80% reduction of *Larp1* gene expression in islets of *β*-*Larp1KO* mice. This is similar to the 80% reduction in mTORC1 signaling found in the *β*-*RaptorKO* mice generated by crossing the *floxed-raptor* mouse with the same *RIP-Cre* mouse used to produce the *β*-*Larp1KO* mice^3^. The RIP-Cre-induced recombination is ~ 90 to 95% of all insulin positive cells^3,4^. We speculate that the remaining 20% expression in mouse islets is coming from non-β-cells, very few cells scaping from recombination and minor acinar contamination of the islet preparation. Due to ectopic cre-recombinase expression outside the pancreas in the *RIP-cre* mice, we confirmed the neutral metabolic phenotype in the *β*-*Larp1KO* mice by using the *Ins1-Cre* mouse. Therefore, it is unlikely that normal β-cell function in the *β*-*Larp1KO* mice is due to poor cre-mediated recombination.

An alternative explanation to the lack of phenotype in the *β*-*Larp1KO* mice would be an up-regulation of other members of the family. The LARP family consists of six members: LARP1, 2 (1B), 4, 5 (4B), 6, and 7^23,25^. They all contain the RNA-binding domain but only LARP1 and LARP1B present the DM15 motif and interact with mTORC1^23,25^. In the *β*-*Larp1KO* mice, islet LARP1B expression was similar to control littermates. Although there was trend to higher levels of LARP6 in *β*-*Larp1KO* mice, this is unlikely to explain the normal phenotype as LARP6 is barely expressed in mouse islets (< 5%) and even a threefold induction would still result in very low levels of LARP6. However, it is possible that expression of other LARP family members at normal or slightly increase levels (LARP6) is sufficient to maintain protein translation and β-cell function.

The small increase in glucose levels observed in the intraperitoneal glucose tolerance test in the *β*-*Larp1KO* mice undergoing long-term high BCAA diet (16 weeks) opens the possibility that the lack of LARP1 potentially limits protein synthesis in prolonged and sustained mTORC1 activation as in diabetogenic conditions. However, the upregulation of LARP1B in diabetes (Fig. 1B) might attenuate the absence of LARP1 in the responses to HFD or BCAA diet. In fact, we did not observe any difference in β-cell mass and plasma insulin in the *β*-*Larp1KO* mice after the BCAA challenge. In human HeLa cells, suppression of LARP1 expression decreased protein synthesis by 15% and caused cell cycle arrest and increased apoptosis^28^. These cells barely express LARP1B (40-fold less than LARP1) and the lack of LARP1B failed to cause phenotypic changes. Therefore, LARP1B is unlikely to be able to compensate for the lack of LARP1 in HeLa cells^28^. Here we show that LARP1 is the most expressed isoform in mouse islets but LARP1B is still expressed (20% compared to LARP1 levels). In human β-cells, the expression of LARP1B is half of LARP1 levels and 63% in diabetic patients (Fig. 1). Although we did not assess LARP1 and LARP1B expression in our diet challenge, it is reasonable to speculate that LARP1B expression in basal and diabetogenic conditions is enough to maintain β-cell function. Indeed, LARP1B has 87% identical sequence within the C-terminal domain that recognizes the TOP sequence and controls 5′TOP mRNA translation^41^. However, LARP1B role in conditions with mTORC1 and C2 inhibition by Torin has been questioned^41^. Future studies deleting both LARP1 and LARP1B are necessary to address this question.

In summary, LARP1 is highly expressed in human β-cells and mouse islets, and is upregulated in diabetes. However, LARP1 is dispensable for pancreatic β-cell function and glucose homeostasis in vivo.

## Methods

### Human β-cells gene expression database

In order to determine LARP1 family gene expression in human β-cells, we assessed publicly available databases^29,30^. LARP family gene expression in human fetal and adult β-cells were calculated from Blodgett et al.^29^. In addition, single-cell transcriptome of healthy and type 2 diabetic subjects’ database by Segerstolpe et al.^30^ was used to assess the expression of LARP family members in β-cells.

### Mice

All the procedures were approved by University of Miami IACUC committee (IACUC protocol #18-168) and performed in accordance with University of Miami Animal Care Policies and the GUIDE for the care and use of laboratory animals. To generate the *floxed-Larp1* mouse, embryonic stem cells containing the floxed *Larp1* construct (Fig. 3A) were obtained from the International Mouse Phenotype Consortium (mousephenotype.org/data/genes/MGI:1890165; Larp1^tm1a (EUCOMM)Hmgu^) and injected into blastocyst to generate chimeric mice by the University of Michigan Transgenic Animal Model core^4^. After identifying germline transmission, founder lines were selected and bred into *C57BL/6J* background. First, *floxed* mice were crossed with mice expressing an enhanced variant of Saccharomyces Cerevisiae FLP1 recombinase (FlpE) in all tissues, under the human β-actin promoter (transgenic B6.Cg-Tg(ACTFlpE)9205Dym/J, available from The Jackson Laboratory, USA, Stock number 005703). This removes both the lacZ and neomycin-resistance cassettes and restores the gene of interest allele containing lox sites flanking exon 4. Mouse LARP1 mRNA has 19 exons. Excising exon 4 will directly disrupts the poly(A)-binding protein recognition domain (PABP) because exon 4 is part of the sequence encoding this region. Moreover, mRNA and protein analysis indicates the lack of exons following exon 4 (exons 5–19) will prevent the translation of the 73 aa La-RNA binding domain (exons 7–9) and the 39 aa Raptor-binding domain (DM15 motif; exons 15–17). To obtain the LARP1 knockout mice specifically in β-cells *(β-Larp1KO*), *floxed-Larp1* mice were crossed with mice expressing cre-recombinase under the rat insulin promoter activity (*RIP-Cre*^*Herr*^ mice)^3,4^. We also disrupted *Larp1* gene by crossing the *Floxed-Larp1* mice with the *Ins1-cre* mouse (B6(Cg)-Ins1tm1.1(cre)Thor/J; Jackson’s lab stock no:026801).

### Metabolic studies

Blood glucose levels were determined from blood obtained from the tail vein using ACCU-CHEK II glucometer (Roche). Glucose tolerance test was performed in 6 h fasted animals by injecting glucose intraperitoneally (2 g/kg). Plasma insulin levels were determined by mouse ultrasensitive specific ELISA (ALPCO) following the manufacturer’s instructions.

### Diets

High Fat Diet (HFD; cat no D12492; 20% of carbohydrate, 20% protein and 60% fat; Research Diets, New Brunswick, NJ) and Branched-chain amino acid enriched diet (BCAA; cat no D07010503; 67% of carbohydrate, 23% protein and 10% fat Research Diets). The BCAA has 150% more leucine, isoleucine and valine concentrations. Control standard diet contains 55% of carbohydrate, 23% protein and 22% fat (ENVIGO).

### Islet isolation and insulin secretion

Islets were isolated by the collagenase method as previously detailed^42^. After injecting 1 mg/ml collagenase P (Sigma) into the common bile, pancreata were digested at 37 °C for 16 min. Islets are separated by Histopaque gradient (Histopaque-1077, Sigma-Aldrich) and recovered overnight in RPMI 1640 (Corning Cellgro) supplemented with 10% FBS, 1% penicillin and streptomycin, and 5.5 mM glucose. Twenty islets per replicate were handpicked and preincubated in 8.0 μm culture plate inserts (Millicell; Merck Millipore) with Krebs buffer containing 125 mM NaCl, 5.9 mM KCl, 2.56 mM CaCl_2_, 1.2 mM MgCl_2_, 25 mM Hepes, 1 mg/ml bovine serum albumin and 2.8 mM glucose for 1 h. After preincubation, islets were moved to a new solution with 2.8 mM glucose for 40 min. Then, islets were moved to buffer containing 16.7 mM for 40 min. After stimulation with high glucose, islets were recovered in acidic ethanol for insulin content assessment. Supernatant and islet lysates in acidic ethanol were stored at − 80 °C for insulin measurement by ELISA (Alpco).

### Quantitative real-time PCR

Total RNA was isolated using RNeasy (Qiagen) from 80 to 100 islets. cDNA was synthesized from 0.5 μg of total RNA using random hexamers and was reverse transcribed using Superscript II (High Capacity cDNA reverse transcription kit; Applied biosystems). Real-time PCR was performed on an ABI 7000 sequence detection system using POWER SYBR-Green PCR Master MIX (Applied Biosystems). Analysis was done by the ΔΔCt method^43^*.* Primers were purchased from IDT Technologies and sequences are shown in Table 1.

**Table 1:** qRT-PCR primer sequences.

Gene name	Sequence
Larp1_ Forward	CAAAAGTGTGCAGCCACAGTC
Larp1_Reverse	CCCATTCTTTTCCTCCCCCG
Larp1B_Forward	TCCCATACAGTCAGGGTGATGA
Larp1B_Reverse	CCAACCTTCTCCACAAGGGG
Larp4_Forward	CCTGCAGGAACCTCGAAAGT
Larp4_Reverse	TGGTTTCTCATGCGGCTTCT
Larp6_Forward	TTCAAGAAGGTGAAACACCTCAC
Larp6_Reverse	GTCCTCGTTCAACTCCAGGG
Larp7_Forward	TGGGCGAGGAGGTTATACCA
Larp7_Reverse	GCCTGCTGTAGGCGCTTTA
INS1_Forward	CACCCCACCTGGAGACCTTA
INS1_Reverse	TGAAACAATGACCTGCTTGCTG
INS2_Forward	GCAAGCAGGAAGGTTATTGTTTCA
INS2_Reverse	GCTTGACAAAAGCCTGGGTG
CycloB_Forward	GGAGATGGCACAGGAGGAA
CycloB_Reverse	GCCCGTAGTGCTTCAGCTT

### β-cell mass quantification

β-cell mass was determined as previously described^3^. After 17 weeks in BCAA diet, animals were euthanized and pancreas carefully removed and weighted. Pancreas were fixed in 4% PFA overnight, immersed in 70% ethanol and embedded in paraffin. Insulin and glucagon cells were stained by overnight incubation with guinea pig anti-insulin (Dako-A0564; 1:400) and mouse anti-glucagon (Abcam-ab10988; 1:500) antibodies, respectively. Fluorescent images were acquired using a microscope (Leica DM5500B) with a motorized stage using a camera (Leica Microsystems, DFC360FX), interfaced with the OASIS-blue PCI controller and controlled by the Surveyor software. β-cell ratio was calculated by measuring insulin and acinar areas using Photoshop software in three insulin-stained sections (5 μm) that were 200 μm apart. To calculate β-cell mass, β-cell to acinar ratio was then multiplied by the pancreas weight.

### Statistics

Data are presented as mean ± SEM. Student t test was employed to assess statistical difference between means of two groups in one time point, e.g. control vs diabetes (Fig. 2C) and control vs *β-Larp1KO* mice (Fig. 3B). One-way analysis of variance (*ANOVA*) followed by Dunnet’s posthoc test was performed to compare LARP family gene expression to the LARP1 mRNA levels (Fig. 2A,B). Two-way *ANOVA* followed by Tukey’s posthoc test was used to identify differences between control vs *β-Larp1KO* mice over time, e.g. body weight gain and glucose levels during intraperitoneal and oral glucose tolerance test. Results were considered statistically significant when the p value was equal or less than 0.05.

## References

[1] Alejandro EU, Gregg B, Blandino-Rosano M, Cras-Meneur C, Bernal-Mizrachi E (2015). Natural history of beta-cell adaptation and failure in type 2 diabetes. Mol. Aspects Med..

[2] Chang-Chen KJ, Mullur R, Bernal-Mizrachi E (2008). Beta-cell failure as a complication of diabetes. Rev. Endocr. Metab. Disord..

[3] Blandino-Rosano M (2017). Loss of mTORC1 signalling impairs beta-cell homeostasis and insulin processing. Nat. Commun..

[4] Alejandro EU (2017). Overexpression of kinase-dead mTOR impairs glucose homeostasis by regulating insulin secretion and not beta-cell mass. Diabetes.

[5] Blandino-Rosano M (2016). 4E-BP2/SH2B1/IRS2 are part of a novel feedback loop that controls beta-cell mass. Diabetes.

[6] Ni Q (2017). Raptor regulates functional maturation of murine beta cells. Nat. Commun..

[7] Helman A (2020). A nutrient-sensing transition at birth triggers glucose-responsive insulin secretion. Cell Metab..

[8] Jaafar R (2019). mTORC1 to AMPK switching underlies beta-cell metabolic plasticity during maturation and diabetes. J. Clin. Invest..

[9] Ardestani A, Lupse B, Kido Y, Leibowitz G, Maedler K (2018). mTORC1 signaling: A double-edged sword in diabetic beta cells. Cell Metab..

[10] Bartolome A (2014). Pancreatic beta-cell failure mediated by mTORC1 hyperactivity and autophagic impairment. Diabetes.

[11] Shimobayashi M, Hall MN (2014). Making new contacts: The mTOR network in metabolism and signalling crosstalk. Nat. Rev. Mol. Cell Biol..

[12] Efeyan A, Comb WC, Sabatini DM (2015). Nutrient-sensing mechanisms and pathways. Nature.

[13] Hinnebusch AG, Ivanov IP, Sonenberg N (2016). Translational control by 5'-untranslated regions of eukaryotic mRNAs. Science.

[14] Thoreen CC (2012). A unifying model for mTORC1-mediated regulation of mRNA translation. Nature.

[15] Jefferies HB (1997). Rapamycin suppresses 5'TOP mRNA translation through inhibition of p70s6k. EMBO J..

[16] Meyuhas O, Kahan T (1849). The race to decipher the top secrets of TOP mRNAs. Biochim. Biophys. Acta.

[17] Terada N (1994). Rapamycin selectively inhibits translation of mRNAs encoding elongation factors and ribosomal proteins. Proc. Natl. Acad. Sci. USA.

[18] Jefferies HB, Reinhard C, Kozma SC, Thomas G (1994). Rapamycin selectively represses translation of the "polypyrimidine tract" mRNA family. Proc. Natl. Acad. Sci. USA.

[19] Fonseca BD (2015). La-related protein 1 (LARP1) represses terminal oligopyrimidine (TOP) mRNA translation downstream of mTOR complex 1 (mTORC1). J. Biol. Chem..

[20] Mura M (2015). LARP1 post-transcriptionally regulates mTOR and contributes to cancer progression. Oncogene.

[21] Deragon JM, Bousquet-Antonelli C (2015). The role of LARP1 in translation and beyond. Wiley Interdiscip. Rev. RNA.

[22] Tcherkezian J (2014). Proteomic analysis of cap-dependent translation identifies LARP1 as a key regulator of 5'TOP mRNA translation. Genes Dev..

[23] Hong S (2017). LARP1 functions as a molecular switch for mTORC1-mediated translation of an essential class of mRNAs. Elife.

[24] Lahr RM (2017). La-related protein 1 (LARP1) binds the mRNA cap, blocking eIF4F assembly on TOP mRNAs. Elife.

[25] Bousquet-Antonelli C, Deragon JM (2009). A comprehensive analysis of the La-motif protein superfamily. RNA.

[26] Berman AJ (2020). Controversies around the function of LARP1. RNA Biol..

[27] Aoki K (2013). LARP1 specifically recognizes the 3' terminus of poly(A) mRNA. FEBS Lett.

[28] Burrows C (2010). The RNA binding protein Larp1 regulates cell division, apoptosis and cell migration. Nucleic Acids Res..

[29] Blodgett DM (2015). Novel observations from next-generation RNA sequencing of highly purified human adult and fetal islet cell subsets. Diabetes.

[30] Segerstolpe A (2016). Single-cell transcriptome profiling of human pancreatic islets in health and Type 2 diabetes. Cell Metab..

[31] Bozadjieva N (2017). Loss of mTORC1 signaling alters pancreatic alpha cell mass and impairs glucagon secretion. J. Clin. Invest..

[32] Sinagoga KL (2017). Distinct roles for the mTOR pathway in postnatal morphogenesis, maturation and function of pancreatic islets. Development.

[33] Stavraka C, Blagden S (2015). The La-related proteins, a family with connections to cancer. Biomolecules.

[34] Condon KJ, Sabatini DM (2019). Nutrient regulation of mTORC1 at a glance. J. Cell Sci..

[35] Yuan T (2017). Reciprocal regulation of mTOR complexes in pancreatic islets from humans with type 2 diabetes. Diabetologia.

[36] Philippe L, Vasseur JJ, Debart F, Thoreen CC (2018). La-related protein 1 (LARP1) repression of TOP mRNA translation is mediated through its cap-binding domain and controlled by an adjacent regulatory region. Nucleic Acids Res..

[37] Xu Z (2017). LARP1 is regulated by the XIST/miR-374a axis and functions as an oncogene in non-small cell lung carcinoma. Oncol. Rep..

[38] Ye L (2016). Overexpression of LARP1 predicts poor prognosis of colorectal cancer and is expected to be a potential therapeutic target. Tumour Biol..

[39] Hopkins TG (2016). The RNA-binding protein LARP1 is a post-transcriptional regulator of survival and tumorigenesis in ovarian cancer. Nucleic Acids Res..

[40] Katsumoto K, Grapin-Botton A (2020). Nutrients men-TOR beta-cells to adulthood. Dev. Cell.

[41] Philippe L, van den Elzen AMG, Watson MJ, Thoreen CC (2020). Global analysis of LARP1 translation targets reveals tunable and dynamic features of 5' TOP motifs. Proc. Natl. Acad. Sci. USA.

[42] Werneck-de-Castro JP, Blandino-Rosano M, Hilfiker-Kleiner D, Bernal-Mizrachi E (2020). Glucose stimulates microRNA-199 expression in murine pancreatic beta-cells. J. Biol. Chem..

[43] Mateus Goncalves L, Pereira E, Werneck de Castro JP, Bernal-Mizrachi E, Almaca J (2020). Islet pericytes convert into profibrotic myofibroblasts in a mouse model of islet vascular fibrosis. Diabetologia.

